# Melatonin and Nitrones As Potential Therapeutic Agents for Stroke

**DOI:** 10.3389/fnagi.2016.00281

**Published:** 2016-11-23

**Authors:** Alejandro Romero, Eva Ramos, Paloma Patiño, Maria J. Oset-Gasque, Francisco López-Muñoz, José Marco-Contelles, María I. Ayuso, Alberto Alcázar

**Affiliations:** ^1^Department of Toxicology and Pharmacology, Faculty of Veterinary Medicine, Complutense University of MadridMadrid, Spain; ^2^Paediatric Unit, La Paz University HospitalMadrid, Spain; ^3^Department of Biochemistry and Molecular Biology II, Faculty of Pharmacy, Complutense University of Madrid, Ciudad UniversitariaMadrid, Spain; ^4^Faculty of Health, Camilo José Cela UniversityMadrid, Spain; ^5^Neuropsychopharmacology Unit, “Hospital 12 de Octubre” Research InstituteMadrid, Spain; ^6^Laboratory of Medicinal Chemistry, Institute of General Organic Chemistry (CSIC)Madrid, Spain; ^7^Neurovascular Research Group, Instituto de Biomedicina de Sevilla, Hospital Virgen del RocíoSevilla, Spain; ^8^Department of Investigation, IRYCIS, Hospital Ramón y CajalMadrid, Spain

**Keywords:** stroke, neuroprotection, oxidative stress, melatonin, nitrones

## Abstract

Stroke is a disease of aging affecting millions of people worldwide, and recombinant tissue-type plasminogen activator (r-tPA) is the only treatment approved. However, r-tPA has a low therapeutic window and secondary effects which limit its beneficial outcome, urging thus the search for new more efficient therapies. Among them, neuroprotection based on melatonin or nitrones, as free radical traps, have arisen as drug candidates due to their strong antioxidant power. In this *Perspective* article, an update on the specific results of the melatonin and several new nitrones are presented.

## Introduction

Stroke is a disease of aging, affecting an increasing number of people worldwide, and the main cause of disability (Flynn et al., 2008; Mathers et al., 2009). The ischemic cascade begins with the energy failure produced by the obstruction of a blood vessel that produces a massive and prolonged release of glutamate (Rothman and Olney, 1986). Physiopathological events associated with brain ischemia are related to oxidative stress process, Ca^2+^ dyshomeostasis, mitochondrial dysfunction, pro-inflammatory mediators and/or programmed neuronal cell death. In the ischemic stroke, as the result of the obstruction of a blood vessel, a critical reduction in the cerebral blood flow (less than 25%) occurred in brain, and neurons need a continued supply of oxygen and glucose. Under deprivation of oxygen and glucose, cell death occurs in two phases: (a) first cell death from anoxia/hypoxia and energy depletion, followed by; and (b) reperfusion that increase oxidative stress and free radical formation, excitotoxicity and nitric oxide (NO) production with ulterior energy failure and delayed death (Hossmann, 1994; Choi, 1996; Lee et al., 1999; Ito et al., 2003).

No effective therapeutic drugs to treat or prevent brain damage in ischemic stroke are currently available. Only recombinant tissue plasminogen activator (r-tPA) is used to open a blood vessel, but r-tPA has a very narrow therapeutic window of 3.5 h (Zivin et al., 1985). Preventing brain damage during the ischemic penumbra, despite that it is a hypoperfused and non-functional tissue, is still a viable tissue adjacent to the infarcted core. Finally, new therapeutic agents are needed to recover tissue functionality before cell death, and to be effective in dealing with several targets, including excitotoxicity and disturbed calcium ion homeostasis, mitochondrial failure, oxidative and nitrosative stress, inflammation and apoptosis (Paschen, 2000; Chan, 2001; Iadecola and Alexander, 2001; Lo E. H. et al., 2005; Niizuma et al., 2010). In this *Perspective* article, we will focus on melatonin and nitrones, well-known radical scavenging and antioxidant agents, for the potential therapy of stroke (Hardeland, 2009).

## Melatonin

Stroke as a main cause of brain disease arouses great interest in therapeutic strategies development. The fact that no effective treatment for stroke has yet been approved to date makes melatonin a promising molecule for stroke treatment, either alone or in combination with other agents. A great number of studies had been developed with melatonin prevention or counteracting stroke damage at several steps of the ischemic cascade, such as neuroinflammation, oxidative stress, excitotoxicity and/or apoptosis (Barlow-Walden et al., 1995; Sinha et al., 2001; Rodriguez et al., 2004; Ozacmak et al., 2009; Reiter et al., 2009; Koh, 2012a; Kim and Lee, 2014; Manchester et al., 2015; Zhao et al., 2015; Alluri et al., 2016). We have recently demonstrated that in rat hippocampal slices subjected to oxygen-glucose-deprivation (OGD) and glutamate excitotoxicity, melatonin is able to mediate neuroprotection (Patiño et al., 2016). Previously, we also demonstrated that melatonin exerts its protective effect post-ischemia through the nicotinic acetylcholine receptor α7 subunit modulated by an overexpression of heme oxygenase-1 (Parada et al., 2014).

Numerous experimental *in vivo* studies evidenced that doses in a range of 5–15 mg/kg of melatonin, mainly administered intraperitoneally, exert neuroprotective effects in the ischemic cascade at several critical points (Guerrero et al., 1997; Pei et al., 2003; Pei and Cheung, 2004; Chen H. Y. et al., 2006; Carloni et al., 2008; Signorini et al., 2009; Balduini et al., 2012; Alonso-Alconada et al., 2013; Paredes et al., 2015).

*In vivo* data confirm the efficacy of this indoleamine. Melatonin has been related to brain repair by comparing pinealectomized and non-pinealectomized animals, observing a greater neurodegeneration in the last group (Manev et al., 1996). Some studies showed its capacity to counteract oxidative stress downregulation or scavenging oxygen and nitrogen species and its free radical detoxification capacity (Guerrero et al., 1997; Pei et al., 2003; Rodriguez et al., 2004; Chen H. Y. et al., 2006; Koh, 2008d).

Other results in stroke models reveal the efficacy of the antiapoptotic properties of melatonin through several mechanisms like increasing levels of Bcl-2, blocking caspase cascade or by preventing mitochondrial depolarization (Sun et al., 2002; Andrabi et al., 2004; Koh, 2008b).

In stroke, elevated extracellular glutamate is critical in neuronal damage. Herein, melatonin has also demonstrated a neuroprotective effect *in vivo*, mitigating Ca^2+^ influx (Camello-Almaraz et al., 2008) via melatonin receptor (Das et al., 2010) by reducing lipid peroxidation (Kim and Kwon, 1999; Wakatsuki et al., 2001). Interestingly, melatonin is a free radical scavenger, which inhibits NO synthesis, a mediator of glutamate and therefore reducing the excitotoxicity (Chung and Han, 2003). In animal stroke models, inflammation leads to numerous pathological events, but melatonin treatment reduces macrophage brain infiltration, activated microglia prevents IL-1β, TNF-α and GFAP overexpression (Lee et al., 2007; Paredes et al., 2015), which taken together inhibit the inflammatory response.

Nonetheless, blood brain barrier (BBB) integrity is compromised after cerebrovascular insults, by an increased release of proinflammatory mediators (COX-2, TNF-α, IL-1β, IL-6), ROS, protein extravasation and interstitial edema. In animal models, melatonin significantly reduces BBB dysfunction through several mechanisms, NO, ROS and RNS levels, preserves tight junction proteins as claudin-5 and modulates hyperpermeability (Chen H. Y. et al., 2006; Grossetete et al., 2009; Song et al., 2014; Moretti et al., 2015; Alluri et al., 2016). In light of these results melatonin shows a suitable profile to preserve BBB functional integrity.

Among brain cell populations, neural stem cells (NSCs) have the potential to regenerate new neuronal population. It has been described that after a melatonin treatment, neurogenesis is induced through melatonin receptor MT2 (Chern et al., 2012). Despite molecular neuroprotective mechanisms are not well defined, melatonin has demonstrated to enhance neurogenic cells of the ischemic brain, in striatum neurons and the hippocampal region (Kilic et al., 2008; Ayao et al., 2010; Lee et al., 2014). Furthermore, mesenchymal stem cells (MSCs) are used in implantations after the ischemic insult, but unfortunately this procedure involves the difficulty that approximately the 80% of the grafted cells do not survive (Roh et al., 2008). Melatonin pre-administration achieves a higher percentage of MSCs survival, also through a receptor-mediated mechanism (Tang et al., 2014).

As far as signal transduction pathways are involved in stroke, melatonin has emerged as a versatile neuroprotective regulator. Melatonin neuroprotective effects are achieved through receptor-mediated mechanisms (MT1, MT2 and MT3; Reiter et al., 2007; Tan et al., 2007; Slominski et al., 2012; Lacoste et al., 2015). Activation of MT1 melatonin receptor leads to the stimulation of a large variety of G proteins (Brydon et al., 1999), upregulation of MT2 promoted neurogenesis and preservation of BBB integrity (Chern et al., 2012) and stimulation of MT3 may contribute to the antioxidant potential of melatonin (Tan et al., 2007).

In addition, melatonin is highly effective in preventing Ca^2+^ dyshomeostasis during ischemic brain injury (Camello-Almaraz et al., 2008; Koh, 2012b). The antiapoptotic effect of melatonin in brain ischemia models is related to its actions at the mitochondria level, preventing the injury-induced reduction of pBad levels and the mitochondrial depolarization inhibiting the mitochondrial permeability transition pore (mPTP; Andrabi et al., 2004; Kilic et al., 2004b; Koh, 2008b). Cell proliferation, differentiation, survival and apoptosis are regulated by the PI3K/Akt signaling pathway and activation of iNOS signaling is associated with PI3K/Akt inhibition. It has been reported that melatonin upregulates this pathway, and decreases iNOS levels (Kilic et al., 2005; Koh, 2008c,d). Matrix metalloproteinases (MMPs) are a family of calcium-dependent zinc-binding proteolytic enzymes that degrade the extracellular matrix (ECM) components of the basement membrane (Montaner et al., 2001). Administration of exogenous melatonin attenuated postischemic MMP-9 activation reducing brain damage in stroke models (Hung et al., 2008). The mitogen-activated protein (MAP) kinase/extracellular-regulated kinase (ERK) 1/2 signaling pathway regulates cell differentiation, growth, survival and apoptosis (Pearson et al., 2001). It has been reported that melatonin plays neuroprotection through Akt and ERK1/2 phosphorylation, and activates MEK/ERK/p90RSK/Bad cascade signaling (Kilic et al., 2005; Koh, 2008a). After hypoxic/ischemic brain injury, endogenous vasoconstrictor endothelin-1 (ET-1) levels are elevated leading to exacerbated brain injury, but when melatonin is administered in mice stroke models a beneficial neuroprotective effect was observed inhibiting ET-1 (Kilic et al., 2004a; Lo A. C. et al., 2005). Phosphorylation/dephosphorylation processes are the major form of cellular signaling (Gong and Iqbal, 2008), and their deregulation turns in severe pathologies including neurodegeneration (Sontag et al., 2004), cancer (Ruediger et al., 2001), cardiovascular (Ling et al., 2012) and metabolic disorders (Mandavia and Sowers, 2012). The phosphoprotein phosphatase 2A (PP2A) is the principal member of the family of Ser/Thr phosphatases (Liu et al., 2005), which removes phosphate from serine and threonine residues. Several compounds may activate or protect PP2A enzyme activity and have neuroprotective actions in *in vivo* and *in vitro* models of brain damage (Shah et al., 2015). In this context, melatonin exerts an upregulation of PP2A enzyme activity, which implies that the PP2A malfunction observed in excitotoxic environments could be mitigated by the administration of melatonin (Koh, 2012a). The protective effect of Silent information regulator 1 (SIRT1) on the brain has been well demonstrated (Yang et al., 2013). Melatonin preserves SIRT1 expression, activates SIRT1 signaling in neuronal cells after hypoxia-ischemia attenuating mitochondrial oxidative damage (Carloni et al., 2014; Yang et al., 2015).

Finally, melatonin has been combined with other drugs, such as t-PA (Chen T. Y. et al., 2006), topiramate (Ozyener et al., 2012), nimodipine and other Ca^2+^ antagonists (Gelmers et al., 1988; Toklu et al., 2009), meloxicam (Gupta et al., 2002) for stroke therapy, giving promising results.

## Nitrones

**Figure 1:**
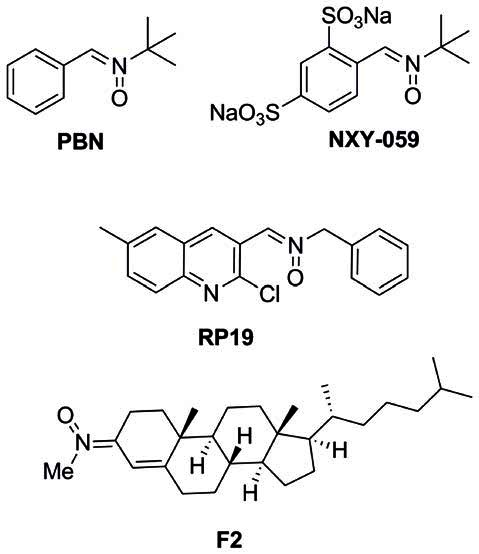
**Structures of nitrones N-t-butylphenylnitrone (PBN), NXY-059, and the nitrones RP19 and F2, assesed in our laboratories**.

Based on the understanding of the biochemical processes involved in the formation and development of a stroke, number of products have been developed targeting the different ischemic and reperfusion events. Despite the promising initial results, neuroprotection drugs for stroke have failed in advanced clinical phases, and consequently, no neuroprotective agent has been approved by the FDA for stroke therapy. However, neuroprotection is still a choice, and oxidative stress, a suitable biological target. In this context, antioxidants such as *N*-*t*-butylphenylnitrone (PBN; Figure 1; Novelli et al., 1986) and NXY-059 (Figure 1; Dehouck et al., 2002), have attracted the interest of a number of laboratories, resulting in therapeutic candidates for cancer (Inoue et al., 2007; Floyd et al., 2008, 2010, 2011; Costa et al., 2015), neurodegenerative disorders (Floyd et al., 2000), hearing loss (Floyd et al., 2008) and stroke (Doggrell, 2006; Floyd et al., 2008). NXY-059 (Figure 1) (Kuroda et al., 1999) is a well-known free radical scavenger with good neuroprotective profile in rat models of transient and permanent focal ischemia, and stroke model in rodents, which has been launched several times in different program in advanced clinical studies, although with limited success (Macleod et al., 2008). In fact and in addition, *tert*-butylnitrones, such as NXY-059, are known to afford *t*-butylhydroxylamines as powerful radical scavengers, after hydrolysis, that further could be oxidized to 2-methyl-2-nitrosopropane which then may synthesize NO radical, the source and origin of the neuroprotection, as it has been already reported for NO donors (Godínez-Rubí et al., 2013). Recently reported new developments have highlighted the not previously described and powerful neuroprotective effect shown by new PBN derivatives bearing *N*-aryl substituents on human neuroblastoma cells, under induced *in vitro* experimental oxidative stress (Matias et al., 2016).

Starting in 2008, the group led by Marco-Contelles (CSIC, Madrid, Spain) has designed, synthesized and developed a number of nitrones for the potential treatment of stroke, the most interesting compounds being RP19 (Figure 1; Chioua et al., 2012), and F2 (Ayuso et al., 2015; Figure 1), in collaboration with Dr. Dimitra J. Hadjipavlou-Litina (Aristotle University of Thessaloniki, Greece) and Dr. Alcázar (Hospital Ramón y Cajal, Madrid, Spain).

As radical-trapping agents, nitrones are expected to delay or prevent oxidation of easily oxidizable substrates, therefore being considered antioxidants. *In vitro* radical trapping and antioxidant activity were studied for nitrones RP19 and F2, and PBN as reference compound, using the DPPH quenching, •OH and ${\text{O}}_{2}^{•-}$ scavenging, and inhibition of lipid peroxidation by AAPH tests. As shown in Table 1A, DPPH and ${\text{O}}_{2}^{•-}$ scavenging activities were low in general, with moderate values for RP19 (42.3% and 23%). Scavenging of •OH, as one of the most toxic radicals generated during ischemic stress, was also determined, showing that higher trapping activities were achieved than reference compound PBN.

**Table 1A:** ***In vitro* antioxidant activity (A) and neuroprotection in neuronal cultures and *in vivo* model of ischemia (B)**.

Nitrone	AAPH (%)^a^ (min)^b^	DPPH (%)^c^	·OH (%)^d^	${\text{O}}_{2}^{•-}$ (%)^e^
RP19^g^	37 (78)	42.3	95	23
F2^h^	55 (nd)^f^	4	83	(nd)^f^
PBN	no (0)	(nd)^f^	90	15

*^a^Determined at 0.1 mM; ^b^Induction time (t_inh_) produced by the tested nitrones; ^c^Determined at 0.5 mM except for F2, that was determined at 0.1 mM; ^d^Determined at 0.1 mM; ^e^Determined at 0.1 mM; ^f^nd: not determined; ^g^Chioua et al. (2012); ^h^Ayuso et al. (2015)*.

Testing in neuronal cultures and in *in vivo* experiments were next evaluated. Thus, the neuroprotective effect of nitrones RP19, F2, as well as PBN and NXY-059 as standards were studied in primary neuronal cultures, which were subjected to OGD as an *in vitro* model of ischemia. Cell viability was evaluated by quantification of living, metabolically active cells, as determined by the MTT assay. Neuroprotection is expressed as the percentage to reach the control value (100%), from the untreated ischemic neurons value (0%) (Table 1B). As shown, all the nitrones tested afforded values in all cases higher than the one determined for PBN (13.4 ± 1.9% at 5 mM) and NXY-059 (56.8 ± 2.5% at 250 μM), being remarkable for those observed for nitrones RP19 (87.5 ± 3.2% at 50 μM) and F2 (80.7 ± 2.7% at 5 μM). Next, transient global brain ischemia was performed on adult rats by the standard four-vessel occlusion model, in which carotid arteries are occluded during 15 min and 24 h after the irreversible occlusion of both vertebral arteries by electrocoagulation (Pulsinelli and Brierley, 1979; García-Bonilla et al., 2007). Ischemic animals were treated with RP19 and F2; NXY-059 diluted in 10% ethanol in saline as a vehicle by intraperitoneal injection when carotid arteries were un-clamped for reperfusion. Animals were studied after 5 days of reperfusion (R5d) after killing by transcardiac perfusion performed under deep anesthesia. Treatments were blindly and randomly performed and body temperature of 37°C was maintained (Table 1B). Cell death and apoptosis were assessed in the hippocampal *cornu ammonis* 1 (CA1) region and cerebral cortex (C). Nitrones RP19 and F2 showed higher inhibition of cell death than for NXY-059. In particular, best results were obtained with F2 at 0.1 mg/Kg concentration, and RP19 at 0.5 mg/Kg concentration. Apoptosis reduction by F2 (35% in hippocampal CA1, 91% in cortex, at 0.1 mg/kg concentration) and RP19 (38% in hippocampal CA1, 79% in cortex, at 0.5 mg/kg concentration) showed the best results, both higher than the values observed for NXY-059 (21% in hippocampal CA1, 55% in cortex), at the same concentration.

**Table 1B:** ***In vitro* antioxidant activity (A) and neuroprotection in neuronal cultures and *in vivo* model of ischemia (B)**.

Nitrone	Cc	Neuroprotection (%)	Time after reperfusion	Cc	Cell death reduction (%)	Apoptosis reduction (%)
PBN	5 mM	13.4 ± 1.9				
NXY-059	250 μM	56.8 ± 2.5	5d	40 mg/kg	17 (CA1) 70 (C)	21 (CA1) 55 (C)
RP19^a^	10 μM	70.9 ± 2.2	5d	0.5 mg/kg	35 (CA1)*** 63 (C)*	38 (CA1)** 79 (C)*
	50 μM	87.5 ± 3.2
F2^b^	1 μM	54.3 ± 1.3	5d	0.05 mg/kg	20 (CA1) 66 (C)	30 (CA1)* 89 (C)*
	5 μM	80.7 ± 2.7	5d	0.1 mg/kg	27 (CA1)** 83 (C)*	35 (CA1)** 91 (C)*

*P < 0.05, **P < 0.01 and ***P < 0.001, compared with vehicle by Dunnett’s post test after ANOVA. ^a^Chioua et al. (2012). ^b^Ayuso et al. (2015).

## Concluding Remarks

In this *Perspective* article, we have updated the current neuroprotection studies and results for the development of melatonin and new nitrones for stroke. Regarding melatonin, the favorable *in vitro* and *in vivo* results reported, together with its great safety profile even at high concentrations, convert this indoleamine into an extraordinary therapeutic option to reduce the multiplicity of effects resulting from the brain ischemic cascade. Unfortunately, there is a lack of clinical studies with melatonin that confirm these results, maybe due to the lack of patentability of this molecule. The most recent results reported by Marco-Contelles’ group confirm that the neuroprotective strategy based on *quinolylnitrones and* *cholesteronitrones* have created great expectations that must still re-confirmed. These nitrones appear as promising agents due to robust antioxidant properties capable to target distinct steps of the biochemical pathways during and after the ischemic insult. Nonetheless, due to the preclinical results reviewed above, the use of melatonin or the new nitrones shown here, or better, multifunctional nitrones bearing the melatonin pharmacophoric motif, may rise as a potential tool to fight against brain ischemia injury and its multiple pathophysiological side-effects.

## Author Contributions

AR and JM-C conceived the present “Perspective”. AR wrote the “melatonin” part, and JM-C wrote the “nitrone” section. MA and AA obtained the unpublished results shown in Table 1B corresponding to the global ischemia experiment of nitrone RP19. MJO-G and FL-M critically read and corrected the manuscript. AR, JM-C, MJO-G and FL-M approved the manuscript.

## Conflict of Interest Statement

The authors declare that the research was conducted in the absence of any commercial or financial relationships that could be construed as a potential conflict of interest.
