# Corrigendum: Melatonin and Nitrones As Potential Therapeutic Agents for Stroke

**DOI:** 10.3389/fnagi.2017.00159

**Published:** 2017-05-29

**Authors:** Alejandro Romero, Eva Ramos, Paloma Patiño, Maria J. Oset-Gasque, Francisco López-Muñoz, José Marco-Contelles, María I. Ayuso, Alberto Alcázar

**Affiliations:** ^1^Department of Toxicology and Pharmacology, Faculty of Veterinary Medicine, Complutense University of MadridMadrid, Spain; ^2^Paediatric Unit, La Paz University HospitalMadrid, Spain; ^3^Department of Biochemistry and Molecular Biology II, Faculty of Pharmacy, Complutense University of Madrid, Ciudad UniversitariaMadrid, Spain; ^4^Faculty of Health, Camilo José Cela UniversityMadrid, Spain; ^5^Neuropsychopharmacology Unit, “Hospital 12 de Octubre” Research InstituteMadrid, Spain; ^6^Laboratory of Medicinal Chemistry, Institute of General Organic Chemistry (CSIC)Madrid, Spain; ^7^Neurovascular Research Group, Instituto de Biomedicina de Sevilla, Hospital Virgen del RocíoSevilla, Spain; ^8^Department of Investigation, IRYCIS, Hospital Ramón y CajalMadrid, Spain

**Keywords:** stroke, neuroprotection, oxidative stress, melatonin, nitrones

María I. Ayuso and Alberto Alcázar were not included as authors in the published article. The authors apologize for this error and state that this does not change the scientific conclusions of the article in any way.

The original article has been updated.

The authors apologize for this oversight. This error does not change the scientific conclusions of the article in any way.

## Conflict of interest statement

The authors declare that the research was conducted in the absence of any commercial or financial relationships that could be construed as a potential conflict of interest.

